# A high propensity for excessive daytime sleepiness independent of lifestyle is associated with cognitive performance in community-dwelling older adults

**DOI:** 10.3389/fpsyt.2023.1190353

**Published:** 2023-08-10

**Authors:** Junxin Wu, Zijing Wu, Caixia Xie, Yongsheng Lin, Zhiqiang Fu, Limao Zhu, Wei Qi, Huali Wang

**Affiliations:** ^1^School of Mental Health, Wenzhou Medical University, Wenzhou, China; ^2^Shangrao Third Hospital, Shangrao, China; ^3^Beijing Dementia Key Lab, Dementia Care and Research Center, Peking University Institute of Mental Health (Sixth Hospital), Beijing, China; ^4^National Health Commission Key Laboratory of Mental Health (Peking University), National Clinical Research Center for Mental Disorders (Peking University Sixth Hospital), Beijing, China

**Keywords:** cognitive function, excessive daytime sleepiness, older adults, community, lifestyle

## Abstract

**Objective:**

The relationship between excessive daytime sleepiness (EDS) and cognitive performance of older adults remains unclear, especially when a healthy lifestyle is considered. The study aimed to explore the association between EDS in passive and active situations and general cognitive function among community-dwelling older adults.

**Methods:**

Two hundred and seventy-one older adults aged 60 and above were recruited from the community cohort in Shangrao. All study participants were free of depression and dementia. The Chinese version of the Epworth Sleepiness Scale (CESS) was used to evaluate EDS. Using the item scores of CESS, the presence of EDS among all study participants were grouped as non-EDS, passive situation-related EDS (PSR-EDS), active situation-related EDS (ASR-EDS), and high sleep propensity (HSP). The Hong Kong Brief Cognitive Test (HKBC) was used to assess cognitive function. Chinese healthy lifestyle metrics were scored based on AHA Life Simple-7. The multivariate logistic regression model was used to estimate the association between the presence of EDS and cognitive function.

**Results:**

The PSR-EDS (*n* = 29, 20.8 ± 5.3) and the HSP groups (*n* = 21, 19.8 ± 4.8) scored lower with HKBC than in the non-EDS group (*n* = 213, 23.2 ± 4.9). The subdomain performance of language in the HSP group was poorer than in the non-EDS group (*p*s < 0.05). Relative to non-EDS, HSP (OR = 3.848, 95% CI = 1.398-10.591) was associated with an increased risk of poor cognitive performance after adjusting age, sex, education, and healthy lifestyle metrics.

**Conclusion:**

High propensity for excessive daytime sleepiness, irrespective of lifestyle, is associated with poorer cognitive performance among community-dwelling older adults. The findings may provide empirical evidence to support sleepiness intervention for reducing the risk of cognitive decline.

## Introduction

In the elderly population, cognitive disorders are very common, which poses a serious challenge to the basic activities of daily life (1). Many factors are related to cognitive function, including education, nutrition, healthcare, lifestyle, mood and sleep (2–4). Sleep disorders are also common conditions in the elderly, characterized by extreme sleep durations, sleep fragmentation, and circadian rhythm disorganization, which may have harmful effects on cognitive function (3, 5–8). Most sleep disorders can be modified and cured (9, 10). Identifying these sleep disorders may provide a chance to prevent cognitive deterioration (2). Previous studies indicate that insufficient sleep might be a predictor of cognitive disorders, such as sleep deprivation, sleep fragmentation, insomnia, as well as short sleep duration (11–14). Recently, mounting evidence linked excessive sleep with cognitive disorders, such as long sleep duration (≥ 10 hours/day) (15–18). Excessive daytime sleepiness (EDS) is characterized by frequent napping during the day, which is another clinical phenotype of excessive sleep (19).

The question that whether EDS is related to cognitive disorders has aroused great interest. Cross-sectional and longitudinal studies have gradually begun to be carried out, but the number of studies remains small. Several cross-sectional studies have reported EDS was associated with cognitive disorders among community-dwelling elderly people (20–23). A study followed 2007 elderly individuals with normal cognition for 10 years and found that EDS increased the risk of cognitive decline (24). Three other studies reached similar conclusions in American population after 3.2–10 years of follow-up (25–27). In addition, several prospective cohort studies have pointed out that EDS or daytime napping was associated with an increased risk of dementia (28–31).

Overall, the available studies indicated that perhaps EDS relates to poor cognitive performance. Nevertheless, patients with EDS reported excessive sleep propensity in different specific situations (32). Dozing in passive situations is generally appropriate while dozing in active situations is usually inappropriate (33). For example, dozing in passive situations may result from a voluntary unopposed sleep drive, when sleep is more welcome than unwanted (e.g., lying down to rest or watching TV). In contrast, dozing in active situations (e.g., conversations) may reflect greater severity and indicate a sleep drive that overwhelms even a strong drive to stay awake. Therefore, individuals who dozed off in the active situations had higher levels of sleepiness than those who dozed off in the passive situations. Namely, perhaps active situations-related EDS are more pathologically significant. Previous studies showed that EDS is associated with poor cognitive performance (20–23, 34). However, few studies classified the severity of EDS based on sleepiness situations and considered the relationship between different levels of EDS and cognitive performance.

In addition, excessive sleep may be predicted by weaker social networks (35). A recent cohort study highlighted that healthy lifestyle including active social contact may be associated with slower memory decline in older adults (36). Will the association between EDS and cognitive performance be altered when a healthy lifestyle is considered? Such question remains unanswered.

Therefore, this study aimed to explore the association between EDS in passive and active situations and general cognitive function among community-dwelling older adults. The association was further examined when the healthy lifestyle was considered. We hypothesized that EDS was independently associated with poorer cognitive performance of older adults.

## Materials and methods

### Participants

We recruited 271 older adults aged 60 and above from the community cohort in Shangrao from March to September 2022. All participants received structured face-to-face interviews from professionally trained investigators. Also, two experienced conducted psychiatric interviews with all study participants to confirm whether the participants were having the clinical diagnosis of dementia or depression. The eligibility criteria for all participants were as follows: (1) age ≥ 60 years; (2) no diagnosis of dementia; and (3) no current diagnosis of depression. The exclusion criteria were as follows: (1) serious physical diseases that prevented completion of the cognitive test; (2) inability to communicate; (3) 9-item Patient Health Questionnaire (PHQ-9) score ≥ 5 (37) or Geriatric Depression Inventory–Rater Interview (GDI–RI) score ≥ 3 (38); (4) 7-item generalized anxiety disorder (GAD-7) score ≥ 10 (39, 40); (5) insomnia severity index (ISI) score ≥ 15 (41); (6) using sedative drugs.

The Ethics Committee of the third people’s Hospital of Shangrao approved this study. All the participants provided informed consent.

### Measures

#### Cognitive performance

Hong Kong Brief Cognitive Test (HKBC) was used as a measure of global cognitive function, which is useful for screening cognitive impairment in older adults and in populations with low educational attainment (42). The HKBC test total score is 30, including immediate recall/attention (1/30), general knowledge (1/30), orientation (5/30), frontal lobe function (2/30), verbal fluency (4/30), delayed recall (8/30), visuospatial construction (3/30), naming and semantic memory (4/30), and recent memory (2/30), with higher scores representing higher cognitive function. Memory was evaluated by the immediate and delayed recall of words for 4 objects and recall of recent news last month; general knowledge was examined by asking for the name of the current leader of the country; orientations included identifying the month, day of the week, season, region, and place name; frontal lobe function was assessed using the Luria 3-step test, in which the subject was asked to first imitate and then complete independently 3 hand motions (fist-edge-palm); verbal fluency was performed by naming animals within 1 min; visuospatial function was assessed using the clock test, including clock-drawing and clock-reading tasks; and naming and semantic memory were tested by asking the participants to name a button and a bicycle tire and then to describe their functions.

#### Excessive daytime sleepiness (EDS)

Excessive daytime sleepiness (EDS) was assessed by the Chinese version of the Epworth Sleepiness Scale (CESS), which assesses sleep propensity in eight situations, namely, situation 1 (sitting and reading), situation 2 (watching TV), situation 3 (sitting inactive in a public place, e.g., a theater or a meeting), situation 4 (as a passenger in a car for an hour without a break), situation 5 (lying down to rest in the afternoon when circumstances permit), situation 6 (sitting and talking to someone), situation 7 (sitting quietly after a lunch without alcohol) and situation 8 (in a car while stopped for a few minutes in the traffic) (43). The frequency of dozing can be divided into “never,” “few,” “more than half,” and “almost all” with scores of 0, 1, 2, and 3, respectively, with a total score of 24. An answer of “more than half” or “almost always” in each situation was defined as having EDS. Situations 1, 2, and 5 are passive (more hypnotic); situations 3, 4, 6, 7, and 8 are active (33).

#### Measurement of depression

Depression was assessed with the 9-item Patient Health Questionnaire (PHQ-9) (37) and Geriatric Depression Inventory–Rater Interview (GDI–RI) (38). Using a cutoff point of 3 to indicate depression, the sensitivity and specificity of the GDI–RI were 93.3 and 87.1%, respectively (38).

#### Chinese healthy lifestyle metrics

We used questionnaire to assess nine Chinese health-related life metrics (including six behavioral metrics and three biological metrics) recently proposed by Jia et al. (36). Behavioral metrics were smoking status (current, past, never), drinking status (current, past, never), physical activity (more than half an hour of physical activity per week), diet (fruit and vegetable consumption), social contact (frequency of attending family/friend gatherings or group activities), and body mass index (calculated from self-reported height and weight, weight/height^2^ in kg/m^2^). Besides, biological metrics contained hypertension, diabetes, and hyperlipidemia. According to the method previously reported to calculate the AHA Life Simple-7 (44), we categorized each behavioral metrics into three levels (coded as poor = 0, intermediate = 1, and optimal = 2) and each of biological metrics into two levels (coded as poor = 0 and optimal = 2). The Chinese healthy lifestyle metrics score ranges from 0 to 18 with higher scores corresponding to better healthy lifestyle (Supplementary Table 1 in the Supplementary material).

#### Covariates

The sociodemographic characteristics assessed included age, sex, marital status, and educational attainment. Educational attainment was classified as follows: illiterate, primary school, secondary school, high school, or junior college or above. In the present study, the comorbidities considered as the co-variates included: hypertension, diabetes and hyperlipidemia, vision impairment, hearing impairment, heart disease, cerebrovascular disease, other chronic organic diseases, and dementia family history. Among these, hypertension, diabetes and hyperlipidemia as biological metrics were included in the Chinese lifestyle metrics. The presence of these comorbidities was binary variables (“Yes” or “No”).

### Statistical analysis

In this study, we analyzed the relationship between common sleepiness situations and cognitive function. Because the proportions of EDS in situation 6 (0.8%) and situation 8 (1.8%) were very low, they were not included in the analysis. Passive situations included situations 1, 2, and 5. Active situations included situations 3, 4, and 7. We calculated the total CESS scores of passive and active situations, respectively. And we used a cutoff point of 4/5 to indicate EDS. Participants with total CESS scores < 5 in both passive and active situations were Non-EDS group (*n* = 213). Participants with a total CESS score ≥ 5 only in passive situations were passive situation-related EDS (PSR-EDS) group (*n* = 29); participants with a total CESS score ≥ 5 only in active situations were active situation-related EDS (ASR-EDS) group (*n* = 8). Participants with total CESS scores ≥ 5 in both passive and active situations were classified in the high sleep propensity (HSP) group (*n* = 21). There were only 8 cases of ASR-EDS, they were excluded from the statistical analysis. Therefore, we mainly analyzed the cognitive performance differences among the other three groups.

Continuous data are displayed as the mean ± standard deviation (SD) or median (interquartile range) based on the distribution of the data. One-Way ANOVA test or Kruskal–Wallis test was used to analyze the differences in the multi-groups. Categorical variables are shown as frequencies or percentages and analyzed with the chi-square test. Logistic regression model was used to explore the associations between comorbidities and cognitive performance.

To determine the relationship between EDS and cognitive performance in elderly people, multiple linear regression model analysis was performed. We used HKBC score as the dependent variable and CESS score, age, sex, educational attainment, and Chinese lifestyle metrics score as independent variables.

Furthermore, we used multivariable logistic regression to explore the associations between EDS subtypes and poor cognitive performance. Individuals with HKBC scores of 21 or lower were considered to have poor cognitive performance; in contrast, those with HKBC scores of 22 or higher were considered to have good cognitive performance. Cognitive function (0 = good cognitive performance; 1 = poor cognitive performance) as a dependent variable, EDS (1 = Non-EDS group; 2 = PSR-EDS group; 3 = HSP group; reference was 1) was an independent variable in the models. Age, sex, educational attainment, other chronic organic diseases, and Chinese healthy lifestyle metrics (≤ 12 = 1; 13–15 = 2; ≥ 16 = 3) were included as covariates. Outcomes were presented as odds ratios (ORs) and 95% CIs after adjustment for confounders. Four different models were tested when EDS was divided into three groups: Model 1 (an unadjusted model), Model 2, Model 3, and Model 4. Model 2 included age, sex, and educational attainment for poor cognitive performance as covariates. Model 3 also included other chronic organic diseases. Model 4 included age, sex, educational attainment, and Chinese lifestyle metrics.

SPSS version 26.0 for Windows (IBM Corp.) was used to conduct all the statistical analyses, and *P* < 0.05 was taken to indicate statistical significance.

## Results

### Demographic characteristics of study participants

The demographic and clinical characteristics of the 263 selected subjects are described in Table 1. There were no significant between-group differences in age, sex, or educational attainment (all *p* > 0.05). The mean lifestyle metrics score in PSR-EDS group was lower than that in Non-EDS group (*p* = 0.043). Furthermore, there were no significant differences in demographic characteristics between men and women in the PSR-EDS and HSP groups, except that women had higher Chinese healthy lifestyle metrics score than men in the HSP group (*p* = 0.021; Supplementary Table 2 in the Supplementary material).

**TABLE 1:** Demographic characteristics, medical history, and healthy lifestyle of study participants.

Variables	PSR-EDS (*n* = 29)	HSP (*n* = 21)	Non-EDS (*n* = 213)	F/χ 2	*p*-value	Non-EDS vs. PSR-EDS	Non-EDS vs. HSP	PSR-EDS vs. HSP
Age, years, mean ± SD	72.2 ± 7.6	70.8 ± 8.1	69.6 ± 7.2	1.633	0.197			
Male, *n* (%)	13 (44.8)	9 (42.9)	87 (40.8)	0.186	0.911			
Married, *n* (%)	21 (72.4)	15 (71.4)	161 (75.6)	0.283	0.868			
Primary school and below, *n* (%)	14 (48.3)	7 (33.3)	57 (26.8)	5.812	0.055			
Vision impairment, *n* (%)	9 (31)	10 (47.6)	37 (17.4)	11.221	0.003	NS	< 0.05	NS
Hearing impairment, *n* (%)	6 (20.7)	3 (14.3)	21 (9.9)	3.377	0.162			
Heart disease, *n* (%)	6 (20.7)	4 (19)	25 (11.7)	2.822	0.25			
Cerebrovascular disease, *n* (%)	1 (3.4)	0 (0)	7 (3.3)	0.358	1			
Other chronic organic diseases, *n* (%)	0 (0)	0 (0)	13 (6.1)	3.21	0.201			
Having depression history, *n* (%)	0 (0)	0 (0)	2 (0.9)	/	1			
Having dementia family history, *n* (%)	2 (6.9)	0 (0)	20 (9.4)	2.29	0.318			
Having surgery history in the past year, *n* (%)	2 (6.9)	1 (4.8)	11 (5.2)	0.166	0.92			
Chinese healthy lifestyle metrics score, mean ± SD	12.6 ± 2.8	12.6 ± 2.9	13.6 ± 2.6	3.21	0.042	0.043	NS	NS

EDS, excessive daytime sleepiness; PSR-EDS, passive situation-related EDS; HSP, high sleep propensity; SD, standard deviation. Categorical variables are expressed as counts (percentages).

### Comparison of the total and item scores of HKBC among three groups

The PSR-EDS (20.8 ± 5.3) and the HSP groups (19.8 ± 4.8) scored lower with HKBC than in the non-EDS group (23.2 ± 4.9) (*p* = 0.015; *p* = 0.002; Table 2). No statistical differences were seen in HKBC item scores between PSR-EDS group and Non-EDS group (all *p* > 0.05). The scores of verbal fluency, naming and semantic memory in the HSP group were significantly lower than those in the Non-EDS group (*p* = 0.024; *p* = 0.015; Table 2).

**TABLE 2:** Comparison of the total and item scores of HKBC in three groups.

HKBC items	PSR-EDS (*n* = 29)	HSP (*n* = 21)	Non-EDS (*n* = 213)	H/F	*p*-value	Non-EDS vs. PSR-EDS	Non-EDS vs. HSP	PSR-EDS vs. HSP
Immediate recall/attention	1 (0, 1)	0 (0, 1)	1 (0, 1)	2.574	0.276			
General knowledge	1 (1, 1)	1 (1, 1)	1 (1, 1)	2.464	0.292			
Orientation	5 (4, 5)	5 (4, 5)	5 (5, 5)	3.258	0.196			
Frontal lobe function	2 (1, 2)	2 (1, 2)	2 (2, 2)	6.930	0.031	NS	NS	NS
Verbal fluency	2 (1, 3)	1 (1, 2)	2 (1.75, 4)	9.205	0.01	NS	0.024	NS
Delayed recall	6 (2.5, 7.5)	4 (2, 6)	6 (4, 8)	7.184	0.028	NS	NS	NS
Visuospatial construction	3 (2, 3)	3 (2, 3)	3 (2, 3)	0.023	0.989			
Naming and semantic memory	3 (3, 4)	3 (3, 3.5)	4 (3, 4)	9.011	0.011	NS	0.015	NS
Recent memory	1 (0, 1)	1 (0, 1)	1 (1, 2)	7.845	0.02	NS	NS	NS
Total HKBC score, mean ± SD	20.8 ± 5.3	19.8 ± 4.9	23.2 ± 4.9	6.940	0.001	0.015	0.002	NS

EDS, excessive daytime sleepiness; PSR-EDS, passive situation-related EDS; HSP, high sleep propensity; SD, standard deviation; HKBC, Hong Kong Brief Cognitive Test. HKBC item scores are expressed as median (interquartile range).

### Factors associated with cognitive performance

Having other chronic organic diseases (OR = 3.28, 95% CI = 1.07–10.09) and high CESS score (≥ 10) (OR = 4.28, 95% CI = 1.92–9.52; Table 3) were associated with poor cognitive performance. The Chinese lifestyle metrics score was positively correlated with HKBC score (β = 0.165, *p* = 0.007; Table 4). Besides, high Chinese lifestyle metrics score (≥ 16) was associated with good cognitive performance (OR = 0.31, 95% CI = 0.15–0.63; Table 3).

**TABLE 3:** Logistic regression on the association between comorbidities and cognitive performance (OR, 95% CI).

Comorbidities	*p*-value	OR	95% CI
Vision impairment	0.301	1.37	0.75–2.5
Hearing impairment	0.067	1.98	0.95–4.13
Heart disease	0.171	1.64	0.81–3.32
Cerebrovascular disease	0.485	0.56	0.11–2.84
Other chronic organic diseases	0.038	3.28	1.07–10.09
Having dementia family history	0.266	0.58	0.22–1.52
Chinese healthy lifestyle metrics score (13–15)	0.981	0.99	0.57–1.74
Chinese healthy lifestyle metrics score (≥ 16)	0.001	0.31	0.15–0.63
CESS score ≥ 10	< 0.001	4.28	1.92–9.52

CESS, the Chinese version of the Epworth Sleepiness Scale.

**TABLE 4:** Linear regression of factors associated with cognitive performance.

Factor	Unstandardized coefficients		Standardized coefficients	t	*p*-value
	B	SE	β		
CESS score	−0.171	0.073	−0.132	−2.333	0.020
age	−0.058	0.040	−0.085	−1.443	0.150
sex	0.198	0.642	0.019	0.309	0.758
education	1.454	0.253	0.343	5.751	0.000
Chinese healthy lifestyle metrics score	0.312	0.115	0.165	2.711	0.007

CESS, the Chinese version of the Epworth Sleepiness Scale.

Chinese version of the Epworth Sleepiness Scale (CESS) score was negatively correlated with HKBC score (β = −0.132, *p* = 0.02; Table 4). As shown in Table 5, PSR-EDS and HSP were associated with poor cognitive performance after adjusting age, sex, education, and other chronic organic diseases (Model 1, 2, and 3 in Table 5). After Chinese healthy life metrics was added in the model (Model 4), the association between HSP and the risk of poor cognitive performance remained significant (OR = 3.848, 95% CI = 1.398–10.591, Model 4, Table 5). However, the association between PSR-EDS and the risk of poor cognitive performance did not survive (OR = 2.225, 95% CI: 0.931–5.316) (Model 4, Table 5).

**TABLE 5:** Logistic regression on the association between different subtypes of EDS and cognitive performance (OR, 95% CI).

	Model 1 (crude)	Model 2	Model 3	Model 4
Non-EDS	Reference			
PSR-EDS	3.087 (1.396, 6.826)	2.447 (1.039, 5.766)	2.716 (1.143, 6.455)	2.225 (0.931, 5.316)
HSP	4.358 (1.682, 11.295)	4.191 (1.514, 11.601)	4.639 (1.664, 12.931)	3.848 (1.398, 10.591)

EDS, excessive daytime sleepiness; PSR-EDS, passive situation-related EDS; HSP, high sleep propensity. Model 2: adjusted for age, sex, and education. Model 3: adjusted for age, sex, education, and other chronic organic diseases. Model 4: adjusted for age, sex, education, and Chinese healthy lifestyle metrics.

## Discussion

Previous studies reported that EDS was independently related to poor cognitive performance (20–23). This study further explored the relationship between EDS in passive and active situations and general cognitive function, especially when healthy lifestyle was taken into considerations. We found that passive situation-related EDS and high sleepiness propensity were associated with an increased risk of poor cognitive performance. The association between high daytime sleepiness propensity and the risk of poor cognitive performance remained significant after adjusting for healthy lifestyle metrics, while the association of passive situation-related excessive daytime sleepiness was marginal.

Individuals were more likely to doze off in passive situations. Napping in passive situations is relatively normal. Therefore, PSR-EDS belongs to the mild subtype. We hypothesize that because the effect of PSR-EDS on cognition is not yet significant, protective factors of cognitive function such as healthy lifestyle can correct its adverse effects. Besides, social engagement may be an important factor in moderating the association between PSR-EDS and poor cognitive performance. Those who are reluctant to participate in social activities and choose to lie down and sleep during the day are more likely to report PSR-EDS. Gooneratne et al. (45) found that EDS among older adults was associated with functional impairments in a broad range of activities, which involve housework, sports, activity all day, and keeping pace with others. Lee et al. (46) also reported that old men with EDS had a low index of social engagement. And poor social engagement increased the risk of cognitive decline in elderly individuals (47).

Individuals with HSP tend to doze in both passive and active situations. Napping in active situations often means that they can not stay awake under a high level of stimulation, which may reflect greater severity of sleepiness (32, 33, 48). In patients with Alzheimer’s disease (AD) and Parkinson’s disease, greater severity of sleepiness was correlated with cognitive deterioration (30, 49). We had similar findings. Compared with older adults without EDS, those with HSP were not only associated with a greater risk of general cognitive difficulties but also had poorer language performance. Compared with PSR-EDS, the uncontrollable sleep tendency of HSP in active situations is more likely to reflect the awakening system dysfunction, which may explain why older adults with HSP had poorer cognitive performance. Research has shown that the functional integrity of the awakening system may be a prerequisite for performing complex cognitive tasks. The awakening system may promote rapid linking between separated cortical regions and have an association with age-related cognitive differences (50, 51). Some of its brain regions also played a key role in emotional and cognitive function (52, 53). We found that healthy lifestyle may be a protective factor for good cognitive performance. Previous studies have also shown that healthy lifestyle is associated with slower cognitive decline and a lower risk of dementia, even in the presence of the APOE ε4 allele (36, 44, 54). However, after controlling for healthy lifestyle metrics, the association between HSP and the risk of poor cognitive performance remained significant. This finding again supported that HSP belongs to the severe subtype of EDS and it’s probably a potential preclinical sign of dementia. Another explanation is that older adults with HSP who dozed frequently in any situation could lack cognitive stimulation. The available studies showed that cognitive stimulation involving group or intellectually challenging activities and social interaction produced benefits on cognition among older adults (55–57). Conversely, it is possible that lacking cognitive stimulation leads to poor cognitive performance.

Overall, we found that EDS was associated with poor cognitive performance. Besides, studies showed that EDS is closely associated with adverse health outcomes and a high incidence of accidents (34, 58–60). EDS not only increases the risk and mortality of metabolic syndrome, coronary heart disease, stroke, and other physical diseases (61–66). Moreover, it is closely related to many neuropsychiatric diseases such as depression, anxiety, Parkinson’s syndrome, and dementia (67, 68). EDS is not a negligible issue that threatens the health of older adults. However, few studies have reported the effective management or intervention of EDS in non-dementia elderly people. We thought it is possible to reduce the attack frequency of EDS by increasing daytime activities such as active social contact, physical exercise, and intellectual activity. Certainly, this hypothesis needs to be confirmed by future studies.

This study has some limitations. First, our measures of EDS in passive and active situations were based mainly on self-reports. It is a more objective and accurate way for evaluating EDS in specific situations to use actigraphy to measure the duration of daytime napping and sleep diaries to record daytime activity. However, this method is complex and difficult to be applied in the community. Second, small sample size and convenience sampling, in particular the small sample size of PSR-EDS and HSP groups. A larger sample of subjects recruited across a broad range of settings is required in future investigations. Third, our assessment of biological metrics was based on self-reported history of diseases. And the evaluation of social contact was relatively single and did not involve social activities such as traveling and chatting online. Fourth, other conditions that might affect EDS, e.g., sleep-related breathing problems, may have not been investigated fully. These should be further examined in future studies.

## Conclusion

In conclusion, the results of this study showed HSP is associated with poorer cognitive performance among community-dwelling older adults. Our results added a piece of evidence that EDS should be included in the management of risk factors for cognitive disorders. Furthermore, the findings may provide empirical evidence to support sleepiness intervention. Probably by encouraging the elderly to maintain healthy lifestyle, participate in social activities and interact with others, they can get the potential benefits of keeping good cognitive performance.

## Data availability statement

The raw data supporting the conclusions of this article will be made available by the authors, without undue reservation.

## Ethics statement

The studies involving human participants were reviewed and approved by the Shangrao Third People’s Hospital. The participants provided their verbal informed consent to participate in this study.

## Author contributions

JW, WQ, and HW contributed to the study’s conception and design. JW, ZW, CX, YL, ZF, and LZ were responsible for data collection. JW was responsible for the data statistics and manuscript writing. WQ and HW provided interpretation of data and revision of the manuscript. All authors contributed to the manuscript revision, read, and approved the submitted version.
