# Supplementary material for: A high propensity for excessive daytime sleepiness independent of lifestyle is associated with cognitive performance in community-dwelling older adults
Source: Front Psychiatry. 2023 Aug 10;14:1190353. doi: 10.3389/fpsyt.2023.1190353 (PMC10448904; doi:10.3389/fpsyt.2023.1190353)
Supplement: Supplementary file 1 [file Table_1.docx]

Table S1. Definition of Chinese healthy lifestyle metrics

| Metrics | Poor level (score=0) | Intermediate level (score=1) | Optimal level (score=2) |
| --- | --- | --- | --- |
| Smoking | Current smoker | Stopped | Never smoked |
| Drinking | Current drinker | Stopped | Never drank |
| Diet (Eat fresh fruits and vegetables) | Sometimes | Often | Every day |
| Physical activity | Never | Three times or less a week | More than 3 times a week |
| Body mass index | ≥30 | 25-29.9 | <25 |
| hypertension | Yes | / | No |
| diabetes | Yes | / | No |
| hyperlipidemia | Yes | / | No |
| social contact | Rarely or never | Occasionally | Often |
