# Supplementary material for: A high propensity for excessive daytime sleepiness independent of lifestyle is associated with cognitive performance in community-dwelling older adults
Source: Front Psychiatry. 2023 Aug 10;14:1190353. doi: 10.3389/fpsyt.2023.1190353 (PMC10448904; doi:10.3389/fpsyt.2023.1190353)
Supplement: Supplementary file 2 [file Table_2.docx]

Table S2. Comparison of the demographic characteristics between men and women in the PSR-EDS and HSP groups.

| Variables | PSR-EDS group | | | HSP group | | |
| --- | --- | --- | --- | --- | --- | --- |
|  | Men  (n=13) | Women (n=16) | P value | Men (n=9) | Women (n=12) | P value |
| Primary school and below, n (%) | 5 (38) | 5 (31) | 0.714 | 2 (22) | 3 (25) | 1 |
| Vision impairment, n (%) | 3 (23) | 6 (38) | 0.454 | 6 (67) | 4 (33) | 0.198 |
| Hearing impairment, n (%) | 1 (8) | 5 (31) | 0.183 | 1 (11) | 2 (17) | 1 |
| Heart disease, n (%) | 1 (8) | 5 (31) | 0.183 | 1 (11) | 3 (25) | 0.603 |
| Cerebrovascular disease, n (%) | 1 (8) | 0 | 0.448 | 0 | 0 | / |
| Having dementia family history, n (%) | 0 | 1 (6) | NS | 0 | 0 | / |
| Having surgery history in the past year, n (%) | 1 (8) | 1 (6) | NS | 0 | 1 (8) | NS |
| Chinese healthy lifestyle metrics score, mean ± SD | 11.5 ± 2.8 | 13.4 ± 2.4 | 0.055 | 11 ± 2.6 | 13.8 ± 2.4 | 0.021* |
| Total HKBC score, mean ± SD | 20.3 ± 5.2 | 21.2 ± 5.5 | 0.645 | 20 ± 4 | 19.6 ± 5.6 | 0.853 |

Based on Fisher's exact test for categorical variables and the t test for continuous variables, * p < 0.05. NS: not significant.
